# Adapted physical activity and hematological malignancies

**DOI:** 10.11604/pamj.2020.37.168.26337

**Published:** 2020-10-16

**Authors:** Nouama Bouanani, Mouna Asly

**Affiliations:** 1 Department of Hematology, University of Mohammed VI of Health Sciences, Casablanca, Morocco,; 2 Department of Physical Medicine and Rehabilitation, University of Mohammed VI of Health Sciences, Casablanca, Morocco

**Keywords:** Hematology, cancer, fatigue, physical activity, supportive therapy

## To the editors of the Pan African Medical Journal

Hematologic malignancies (HM) are a heterogeneous group of neoplasms of clonal origin, originating from hematopoietic tissues; it includes leukemias, lymphomas, myelomas and myeloproliferative and myelodysplastic syndromes. They represent 6.5% of all cancers worldwide and more than 50% of affected patients are over 65 years of age [1,2]. In Morocco, there are no national cancer registries to establish-precise statistics. Nonetheless, according to the cancer registry of Casablanca, malignant lymphomas and leukemia represent 5.8% and 2.3% of all cancer cases recorded between 2008 and 2012, respectively [3].

Treatment is based on heavy therapies including high-dose chemotherapy, targeted therapy, immunosuppressive treatment, bone marrow transplant, which could be carried out depending on the pathology, in an outpatient clinic, day hospital, or in a conventional hospitalization. The average length of stay is 3-4 weeks for acute induction leukemia and bone marrow transplants. It is well established that cancer and its treatment cause of multiple acute and chronic side effects such as haematological toxicity (anemia, febrile aplasia and hemorrhagic syndrome), digestive complications (diarrhea, vomiting and mucositis), neurological disorders (neuropathy peripheral and balance disturbances), toxic cardiomyopathy due to antracyclines with a decrease in left ventricular ejection fraction, thromboembolic complications, renal toxicity by platinum salts and ifosfamide, hearing, skin and gonadal. Fatigue, of multifactorial origin, is a symptom frequently reported by patients with malignant hemopathies. It occurs in the early stages of treatment and persists after stopping it. It is not reduced by sleep or rest [4].

Amyotrophy and cardio-respiratory deconditioning, secondary to intensive treatment, prolonged bed rest and confinement, promote reduced physical performance and sustain fatigue persistence. A change in body composition is also observed in patients with malignant hemopathies with increased body fat at the expense of lean body mass [5]. Psychic disturbances, such as depression, anxiety and sleep disorders are frequently associated with hematologic malignancies, with a prevalence varying between 37% and 47% depending on the series [5,6]. Such disorders adversely affect the quality of life of these patients. Physical activity is a non-pharmacologic therapy that may provide substantial health benefits for cancer patients, reducing the symptoms and side effects of cancer. It can be integrated throughout the entire patient's care path: in prevention, during and after treatment for malignant hematologic disease.

Numerous studies have demonstrated the feasibility, safety and benefit of physical activity in different types of hematologic malignancies, or during hematopoietic stem cell transplant procedures. They report an improvement in individual physical performances, reduction in fatigue and depression and improvement in the quality of life [7,8]. The objectives of the practice of physical activity in patients with malignant hemopathies are to fight, early, against sedentarity and to maintain an active lifestyle, irrespective of the disease stage. During treatment, functional rehabilitation may be offered, aiming at maintaining the patient's physical activity, while respecting his clinical condition and the side effects of treatment.

After treatment, interventional programs based on aerobic endurance exercises and muscle strengthening exercises should be favored [9]. This program can be structured as following: practice at least 30 minutes of physical activity per day of a moderate intensity cardio-respiratory type (walking at high speed, running and cycling), at least 5 days/week; practice at least two muscle strengthening sessions per week, lower limbs, upper limbs and trunk, respecting 1 to 2 days of recovery between each session; perform stretching joint flexibility and mobility exercises 2 to 3 times a week; incorporate balance exercises for people aged 65 and over, at least twice a week; recommend at least 1 hour of moderate to high intensity physical activity every day, at least 3 days a week, for children and teenagers from 6 to 17 years old.

Personalized and adapted programs must be offered, taking into account patient´s specificities and the course of the disease. Consequently, an initial clinical evaluation is necessary to identify the absolute contraindications to the physical activity practice, specify the previous level of activity and assess the patient´s physical aptitudes. Physical activity program, assisted or in total autonomy, requires observance of basic safety rules, respect for the different phases of physical activity practice (warm-up, activity, recovery and stretching) and control of tolerance.

## Conclusion

Physical activity is an essential supportive care in the management of patients with malignant hemopathies. The practice of adapted physical activity contributes to the improvement of the quality of life of patients within the framework of a multidisciplinary care. Raising patient awareness of the benefits of physical activity is essential, as it should be integrated as early as possible in the care plan.
